# Non-uniform Segmental Range of Motion of the Thoracic Spine During Maximal Inspiration and Exhalation in Healthy Subjects

**DOI:** 10.3389/fmed.2021.699357

**Published:** 2021-08-30

**Authors:** Jesús Burgos, Carlos Barrios, Gonzalo Mariscal, Alejandro Lorente, Rafael Lorente

**Affiliations:** ^1^Division of Pediatric Orthopedics, Hospital Ramon y Cajal, Madrid, Spain; ^2^Institute for Research on Musculoskeletal Disorders, Valencia Catholic University, Valencia, Spain; ^3^Orthopaedic Surgery Department, Hospital Ramon y Cajal, Madrid, Spain; ^4^Spine Surgery Unit, Hospital Infanta Cristina, Badajoz, Spain

**Keywords:** thoracic spine, respiratory dynamics, forced ventilation, inspiration and active expiration, kyphotic angle

## Abstract

**Background and Objective:** To analyse the range of motion of the thoracic spine by radiographically measuring changes in the sagittal profile of different thoracic segments during maximal inspiration and exhalation. The starting hypothesis was that forced deep breathing requires an active, but non-uniform widening of the lordotic–kyphotic range of motion of the different thoracic segments.

**Methods:** Cross-sectional study. Participants were 40 healthy volunteers aged 21–60. Conventional anteroposterior and functional sagittal chest radiographs were performed during maximal inspiration and exhalation. The range of motion of each spinal thoracic functional segment, global T1–T12 motion, and the sagittal displacement of the thoracic column during breathing were measured. Considering the different type of ribs and their attachment the spine and sternum, thoracic segments were grouped in T1–T7, T7–T10, and T10–T12. The displacement of the thoracic spine with respect to the sternum and manubrium was also recorded.

**Results:** The mean difference from inspiration to exhalation in the T1–T12 physiologic kyphosis was 15.9° ± 4.6°, reflecting the flexibility of the thoracic spine during deep breathing (30.2%). The range of motion was wider in the caudal hemicurve than in the cranial hemicurve, indicating more flexibility of the caudal component of the thoracic kyphosis. A wide range of motion from inspiration to exhalation was found at T7–T10, responsible for 73% of T1–T12 sagittal movement. When the sample was stratified according to age ranges (20–30, 30–45, and 45–60 yr.), none of the measurements for inspiration or exhalation showed statistically significant differences.

Only changes at this level showed a positive correlation with changes in the global thoracic kyphosis (*r* = 0.794, *p* <0.001).

**Conclusion:** The range of motion of the thoracic spine plays a relevant role in respiration dynamics. Maximal inspiration appears to be highly dependent on the angular movements of the T7–T10 segment.

## Introduction

There is no evidence of any functional active participation of the thoracic spine in respiratory mechanics. However, diverse alterations of respiratory function have been described in patients with spinal disorders such as scoliosis and hyperkyphosis. Such studies have addressed only ventilatory and gas exchange, overlooking respiratory mechanical movements. Restrictions in the range of motion (ROM) imposed by rib attachments establish the thoracic spine as a passive stabilizer during breathing movements. Conversely, the diaphragm, the intercostals, and other accessory respiratory muscles have active and determinant roles in respiratory function (1, 2).

During inspiration, the ribs move up and expand laterally, increasing the anterior–posterior and lateral diameter of the thoracic cage. In contrast, the ribs move down and medially during exhalation. In these vertical movements, the thoracic spine is considered to have a restricted ROM because of its anatomic skeletal junctions. The sternum and ribs display a continuous and harmonious mobility during breathing, representing a significant participation in respiratory dynamics (3). These bony structures are, therefore, components of the mobile chest area during breathing. However, the role of the thoracic spine in respiratory function has so far been poorly studied and is not understood.

A previous study has contended that the thoracic spine is fixed during tidal breathing as the pivot of the ribcage (4). However, the functional ROM of the thoracic spine on the sagittal plane is wider than expected. Recently, a mean ROM of 31.7° was measured in 50 healthy adults via CT scanning of the thoracic spine in forced flexion and extension (5). Previous studies have analyzed the movements of the thoracic cage during breathing (6–8), but none have measured angular variations at thoracic segmental levels on the sagittal plane, especially during deep breathing. Our hypothesis was that the thoracic spine ROM has a relevant role in respiratory mechanics during maximal inspiration and exhalation. Support of this hypothesis would lead to several important clinical implications especially for the understanding of respiratory limitations in patients with spinal deformities, as well as for the surgical treatment of these patients requiring thoracic fusion.

Therefore, the purpose of this study was to analyze the ROM of the thoracic spine during forced respiratory dynamics. Changes in the sagittal profile of the thoracic spine during maximal inspiration and exhalation movements were radiographically measured at all spinal segments, and spatial relationships between the thoracic spine and the sternum during deep breathing were evaluated. This work proposes a new set of radiographic parameters that could be incorporated into the clinical analysis of respiratory function, particularly in patients with thoracic spine diseases.

## Materials and Methods

### Ethics Statement

The study protocol was approved by the Institutional Review Board of the Hospital Ramón y Cajal (Madrid, Spain) with protocol number: V1-08/05/2016. All participants were informed about the purpose of the research and were asked to sign a consent form before taking part in the study. Procedures were in accordance with the ethical standards of the national ethics guidelines for research involving humans and with the Helsinki Declaration revised in 1983. Confidentiality was ensured during data collection and subsequent publication of the results.

### Participants

This study was conducted in 40 healthy adult volunteers, 23 women and 17 men ranging in age from 21 to 60 years, after proper informed consent was obtained. Before the radiological exam, all subjects were screened to ensure no known history of back pain, lung disease, heart disease, or any other health problem that might have affected the study. Exclusion criteria were the presence of severe anterior osteophytes and degenerative discopathies and thoracic kyphoscoliosis as well as an inaccurate quality of the radiographic exams which complicates the measurement of all radiological parameters included in the study. Table 1 shows the anthropometric characteristics of the sample.

**Table 1:** Sample characteristics.

	**Total sample *n* = 40**
Age (years)	40.8 ± 13.6
Sex (Male/Female)	17/23
Weight (kg)	71.7 ± 13.3
Height (cm)	168.1 ± 18.0
BMI (kg/m^2^)	24.0 ± 3.3

*BMI, body mass index*.

### Radiographic Measurements

Conventional anteroposterior and functional sagittal chest radiographs were performed during the maximal inspiratory and expiratory phases. The anteroposterior radiographs were used to identify the number of ribs per participant and to rule out previous vertebral or pulmonary pathologies, whereas the functional sagittal radiographs were used to measure the different parameters included in this study.

In all cases, the same conventional procedure for obtaining lateral radiographs of the thoracic in standing position was carefully respected by the same technicians at the hospital radiology department. The patients were instructed to look straight ahead with the chin up maintaining their arms raised over the shoulder level by holding the hands to a support incorporated to the image detector panel. The left side of the thorax was positioned against the image receptor to minimize cardiac magnification. The central ray was always centered at the level of the T7 vertebra. The patient was first asked to take a deep breath and hold the breath while the exposure for maximal inspiration was taken. Afterwards, the patients were asked to perform and maintain for a few second a maximal exhalation to take the second lateral radiograph.

Digital images were measured in a computer by using the Surgimap Spine software (Nemaris Inc., Methuen, Massachusetts, USA). Two spine surgeon specialists with extensive experience in the measurement of sagittal profile of the thoracic spine in radiographs perform the measurements. Often there were difficulties to visualize the upper thoracic levels (T1–T3) and therefore impossibility for measuring kyphotic angles. In such cases our protocol was first to change the characteristics of the digitalized image by the acquisition software until the anatomic bony structures were satisfactorily visible and measurements could be registered. In case of failure, the radiographs were excluded from the study.

**Figure 1:**
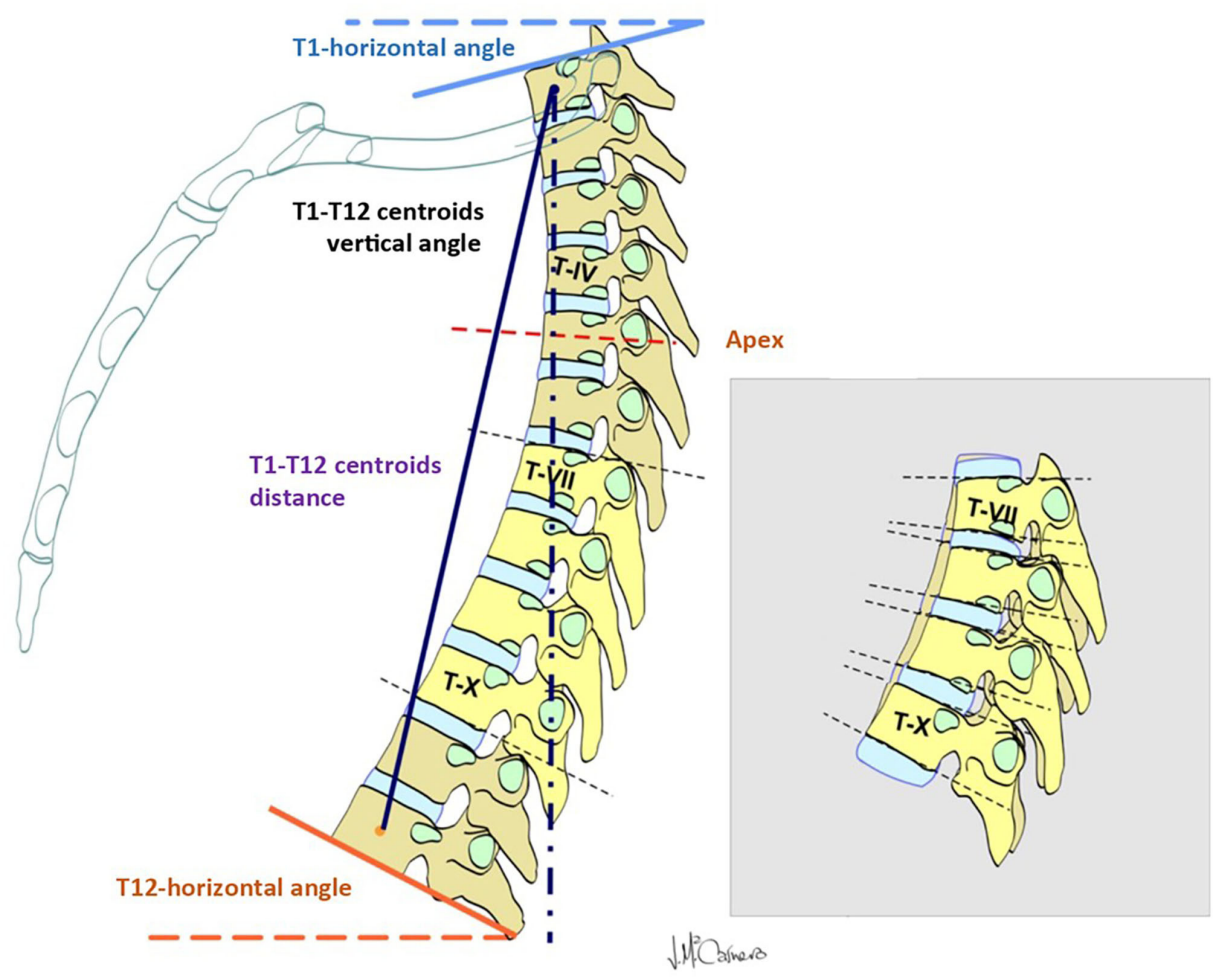
Parameters measured to evaluate the mobility of the thoracic spine.

**Figure 2:**
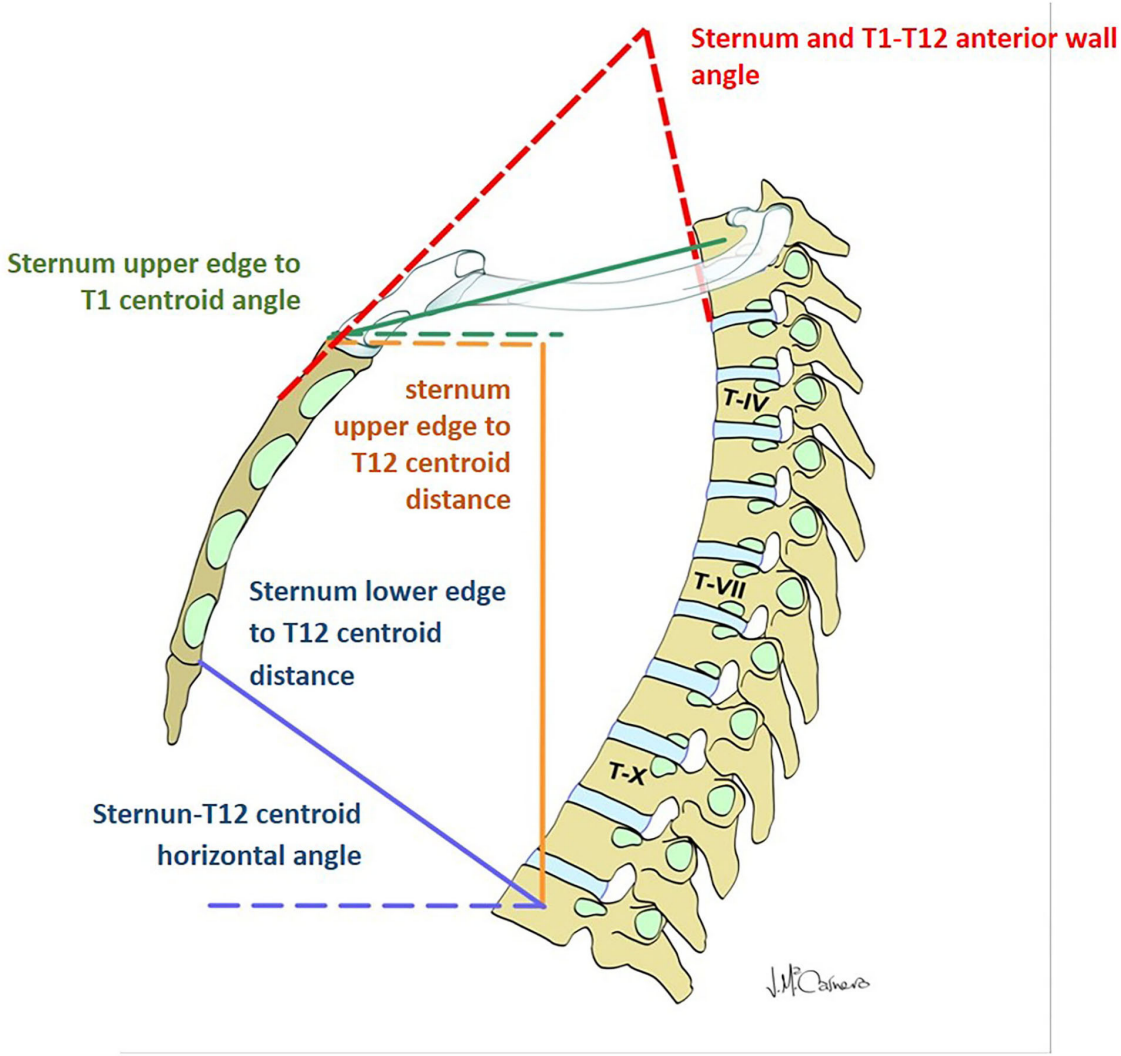
Parameters evaluating the displacement of the thoracic spine with respect to the sternum and manubrium.

The first group of radiological parameters assessed the displacement of the thoracic spine during inspiration and exhalation, which indicates the mobility of the thoracic spine (Table 2; Figure 1). The ROM of each functional thoracic spine segment was measured by the angle that formed between the proximal and the distal endplates of the adjacent vertebral bodies (9). Results were also grouped in three spine sectors (T1–T7, T7–T10, and T10–T12) corresponding to the three anatomic rib attachments. The ROM of the global T1–T12 ROM was also measured. The second group of parameters intended to evaluate the relationship of the thoracic spine to the sternum during the breathing process (Table 2; Figure 2).

**Table 2:** Definition of the radiological parameters assessed in this study.

**Parameters**	**Definition**
**I. Mobility of the thoracic spine**	
T1–T12 thoracic kyphosis angle	Angle measured from the T1 cranial or cephalic epiphyseal plate to the T12 caudal epiphyseal plate. This parameter assesses how the thoracic spine behaves during maximal inspiration and exhalation movements, showing the T1–T12 global ROM.
Kyphosis angle of the cephalic thoracic hemicurve	Angle from the T1 cranial endplate to the bisector of the endplates of the apex vertebra of the physiologic thoracic kyphosis.
Kyphosis of the caudal thoracic hemicurve	Angle between the previous bisector of the apex vertebra of the kyphosis to the T12 caudal endplate.
Apex	Apex level of the kyphosis at maximal inspiration and exhalation.
T12 distal endpate angle	The angle between the caudal T12 endplate and the horizontal. This angle monitors changes in the inclination of the last vertebra of the thoracic spine during peak respiratory movements.
T1 proximal endplate angle	The angle between the proximal or cephalic T1 endplate and the horizontal. This angle identifies the spatial displacement of the T1 vertebra, that is, the proximal end of the thoracic spine, during peak respiratory movements.
T1–T12 centroid distance	The distance from the centroid of the T1 vertebra to that of the T12 vertebra. This measurement indicates changes in the height of the thoracic spine at maximal inspiration and exhalation.
T1–T12 centroids vertical angle	The angle formed between the line of the T1–T12 centroids and the vertical line at the T1 centroid. This parameter shows the spatial displacement of the thoracic spine on the sagittal plane with respect to the vertical axis from inspiration to exhalation.
**II. Displacement of the thoracic spine with respect to the sternum and manubrium**	
Sternum T1–T2 angle	The angle of the anterior cephalic border of the sternum with the line joining the anterior edges of the T1 and T2 vertebral bodies.
Inferior distance sternun/T12	The distance from the posterior–inferior edge of the sternum to the T12 centroid.
Lower sternun slopping	The angle of the horizontal with the line formed by the posterior–inferior edge of the sternum to the T12 centroid.
Anterior-superior distance sternun/T1	The distance from the anterior–superior edge of the sternum to the T1 centroid.
Upper sternun slopping	The angle of the horizontal with the line formed by the anterior–superior edge of the sternum and the T1 centroid.
Distance T12 to horizontal sternum line	The vertical distance from the T12 centroid to the horizontal line from the anterior–superior end of the sternum.

### Statistical Analysis

Statistical analyses were performed using the SPSS, version 21 (IBM Corp., Armonk, NY). As all measurements comprise continuous numeric data, descriptive results are expressed as the mean ± standard deviation (SD). Based on the limited sample, changes between maximal inspiration and exhalation were analyzed using the non-parametric paired Wilcoxon signed-rank test. To assess differences in thoracic spine stiffness related to age, patients were divided into three age ranges: 23–30, 30–45, and 45–60 yr. Differences between the three age groups was analyzed by the Kruskal-Wallis test. The possible correlations between different radiological measurements were tested by the Spearman' *rho*. Results with a *p*-value ≤ 0.05 were considered as statistically significant.

## Results

### Mobility of the Thoracic Spine

Table 3 shows the mean ± SD values of all parameters reflecting the ROM of the thoracic spine during breathing. Statistically significant differences between inspiration and exhalation were observed in all evaluated parameters. The mean difference from inspiration to exhalation in the T1–T12 physiologic kyphosis was 15.9° ± 4.6° (a range of 9°-27° Cobb), reflecting the relatively high flexibility of the thoracic spine (30.2%).

**Table 3:** Movements of the thoracic spine on the sagittal plane.

	**Inspiration**		**Exhalation**		**ROM**		**Wilcoxon signed-rank test**	
	**Mean ± SD**	**Range**	**Mean ± SD**	**Range**	**Mean ± SD**	**Range**	**Z**	***p***
T1–T12 kyphosis (degrees)	36.8 ± 7.3	25–50	52.6 ± 7.6	37–66	15.9 ± 4.6	9–27	−5,513	0.000
Upper hemicurve (degrees)	15.8 ± 4.8	10–28	21.9 ± 5.0	11–34	6.1 ± 3.3	0–15	−5,096	0.000
Lower hemicurve (degrees)	21.0 ± 5.0	11–29	30.8 ± 5.2	22–45	10.2 ± 4.0	5–22	−5,449	0.000
Apex level	5.4 ± 1.1	3–7	6.7 ± 1.0	4–8	1.3 ± 0.5	1–3	−5,742	0.000
T12 angle *h* (degrees)	21.2 ± 6.7	8–34	24.5 ± 6.2	11–36	3.6 ± 2.9	0–10	−4,683	0.000
T1 angle *h* (degrees)	15.4 ± 6.6	6–28	26.1 ± 6.8	15–39	11.1 ± 4.8	1–22	−5,446	0.000
T1–T12 centroid (mm)	293.1 ± 24.7	245–341	281.9 ± 24.8	228–336	13.2 ± 2.9	6–18	−4,822	0.000
T1–T12 line angle (degrees)	22.9 ± 6.4	11–37	41.4 ± 8.3	24–57	18.5 ± 6.1	4–29	−5,514	0.000

**Figure 3:**
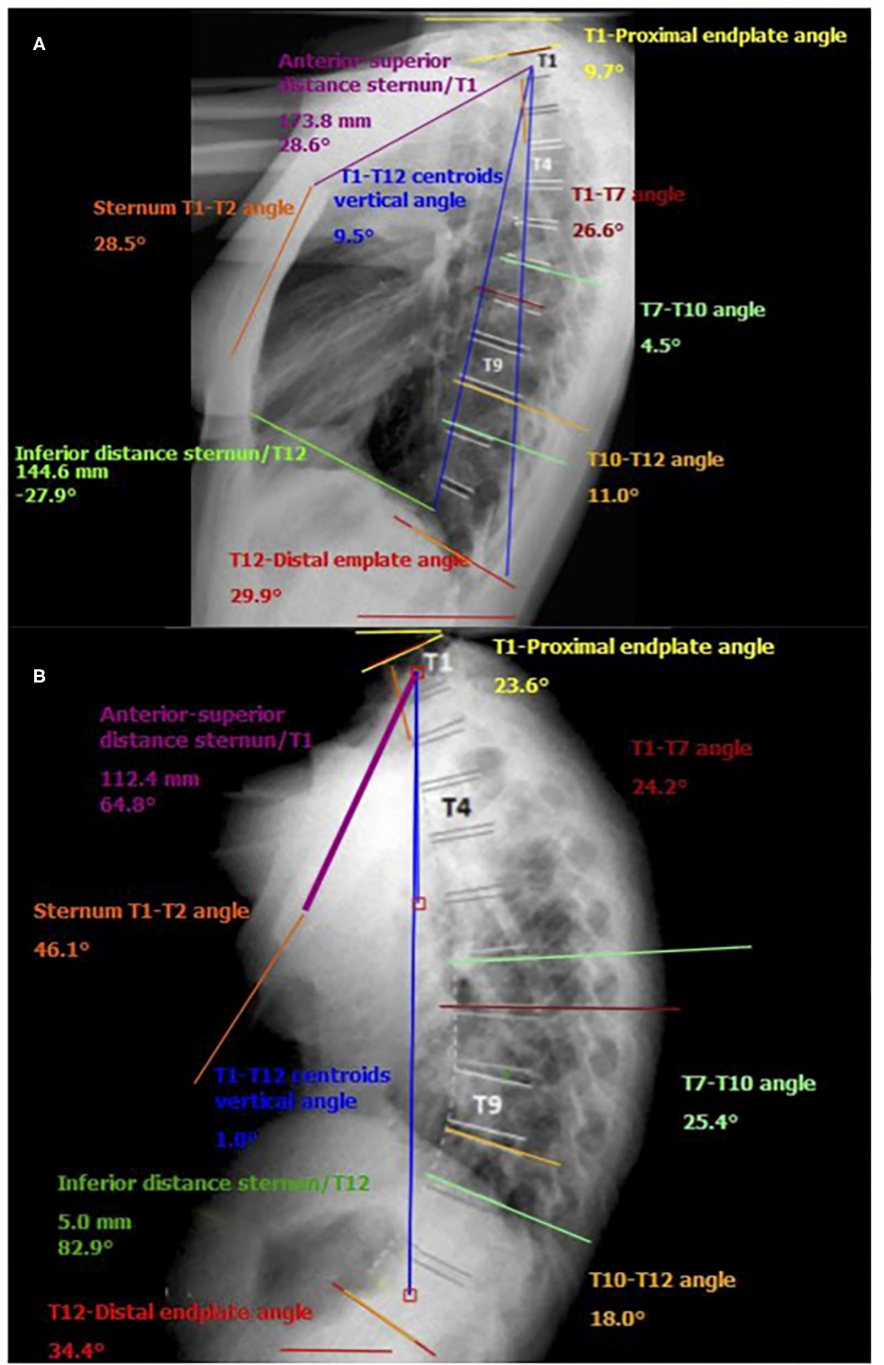
Example of measurements taken by the surgimap software from the lateral radiographs of the thoracic spine in one of the participants. **(A)** forced inspiration; **(B)** forced exhalation.

The apex of the expiratory thoracic kyphosis descended 1.3 ± 0.5 levels from that at inspiration. This change is attributable to the asymmetrical motion of the cranial and caudal hemicurves, and it is related to their different degrees of stiffness. The inclination of the T12 and T1 vertebrae from the horizontal increased at exhalation, being higher at the upper levels, with a mean ROM of 11.1° ± 4.8° for T1 vs. 3.6° ± 2.9° for T12. Figure 3 shows some the measurements taken by the surgimap software from the lateral radiographs of the thoracic spine in one of the cases. Changes in T1–T7, T7–T10, and T10–T12 from inspiration to exhalation are clearly apparent.

From exhalation to forced inspiration, the increment in the distance from the T1 centroid to the T12 centroid was 13.2 ± 2.9 mm (4.7%). The angle of the line joining the T1 and T12 centroids with the vertical showed a relevant mean increment of 18.5° ± 6.1° during exhalation, indicating a displacement in the flexion of the thoracic spine. When the sample was stratified according to age range, none of the measurements for inspiration or exhalation showed statistically significant differences.

**Figure 4:**
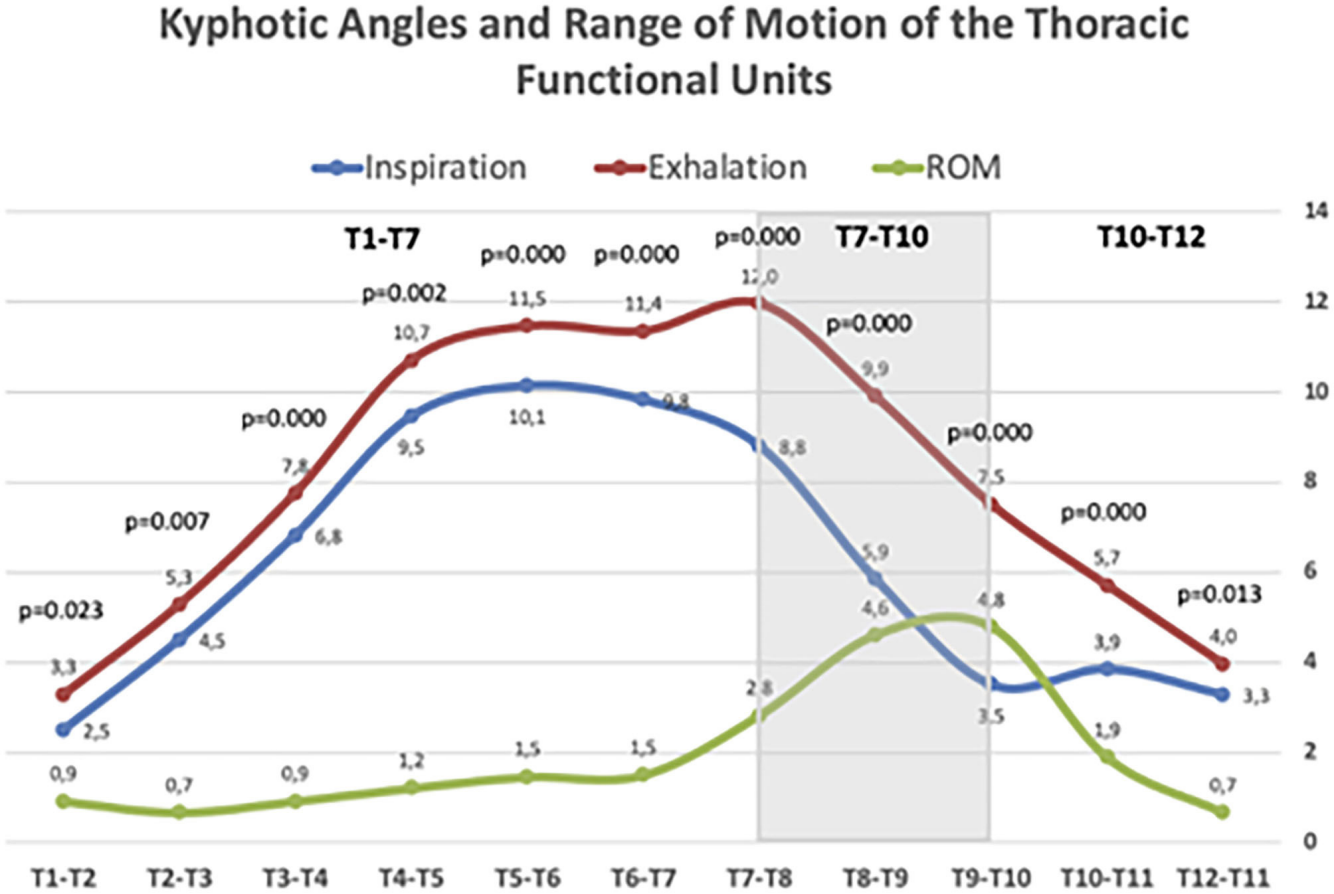
Kyphotic angles and Range of motion (ROM) of thoracic spine segments during maximal forced inspiratory and expiratory phases. Values age given in mean Cobb angle. Statistically significant differences between inspiratory and expiratory angles are given for each segment according to the Wilcoxon rank test.

Figure 4 illustrates the ROM of the functional spinal units of the thoracic spine. In all disc levels, changes from forced inspiration to exhalation were statistically significant. The T4–T7 segment, corresponding to the central thoracic, conform the greatest part of the physiological kyphosis; however, the discs included in this segment showed little change, but still statistically significant, during inspiration and exhalation. Large differences in the ROM from inspiration and exhalation were observed at the discs of the T7–T10 segment. At this level, the mean ROM of the three discs involved was 4.1°, higher than at upper levels (mean ROM of the discs T1–T7 = 1.1°). The discs of the T10–T12 segment (theoretically the most flexible in the thoracic spine) displayed almost similar mobility during forced respiratory movements (mean ROM = 1.3°). The T1–T3 segment showed minimal respiratory mobility, indicating greater stiffness in the thoracic spine.

**Figure 5:**
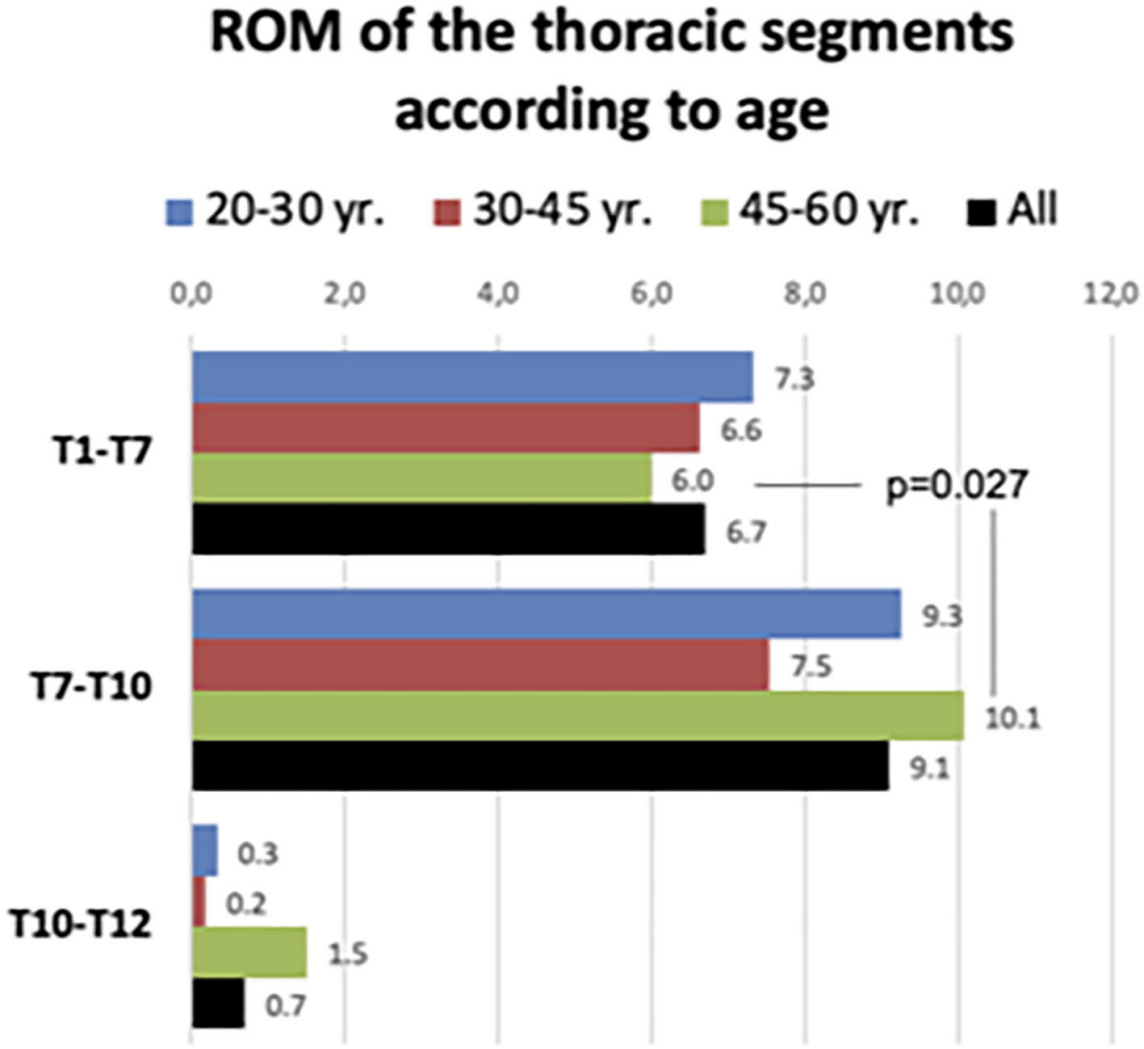
Range of motion of the three main thoracic segments in the whole sample and according to age range.

The compiled ROM of the T1–T7, T7–T10, and T0–T12 segments are given in Figure 5. The global ROM during deep breathing in each thoracic sector is presented both for the whole sample and by discriminating the three age ranges (20–30, 30–45, and 45–60 yr.). There no statistically significant differences in ROM among the three age ranges in any of the thoracic segments. In the three age ranges, ROM of T7–T10 was greater than that found at T1–T7 segment, but differences were not statistically significant, except for the older participants. In this older age range, T7–T10 ROM was greater that T1–T7 ROM (Wilcoxon rank test: Z = 2.205; *p* = 0.027) (Figure 5).

Overall, the ranges of motion of the three discs of the T7–T10 segment were responsible for 74% of the T1–T12 sagittal movement. This figure slightly decreases as increases the age range: in younger participants (20–30 yr.) the contribution of the T7–T10 segment to the total T1–T12 ROM was 78.5%; in the age range 30–45 yr. was 72.8% and in the older participants 69.8%. However, the differences were not statistically significant.

**Figure 6:**
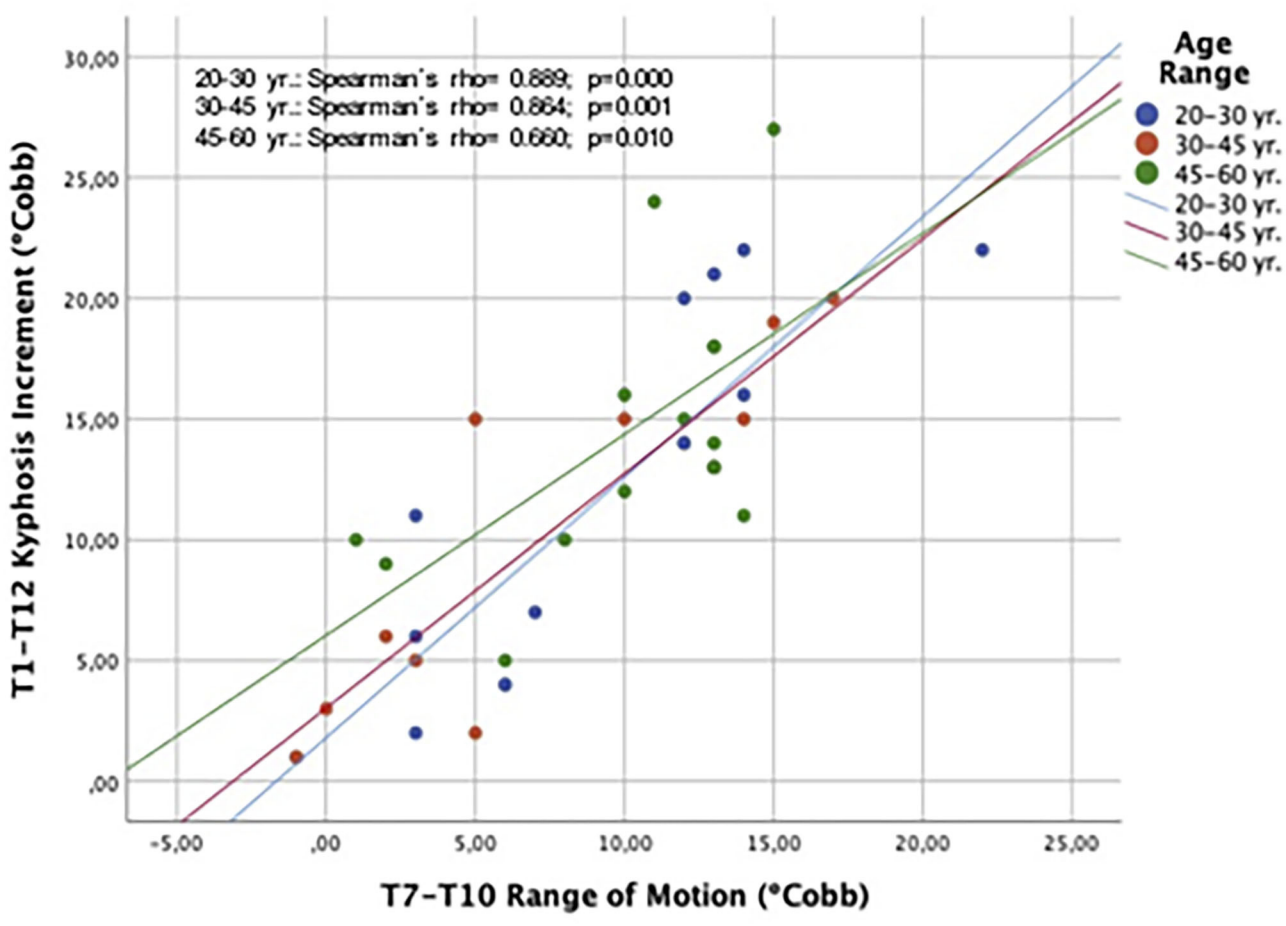
Spearman's correlation plot showing the relationship between changes in T7–T10 ROM and the global T1–T12 kyphosis during forced respiratory movements. Results are discriminated by age ranges.

An analysis of the contribution of the three main segments to the global T1–T12 kyphosis that only changes in the ROM of the T7–T10 segment showed a positive correlation with changes in the global thoracic kyphosis (Spearman's *rho* = 0.794, *p* <0.001). Figure 6 shows that the correlation between T7–T10 ROM and the change of global T1–T12 kyphosis applied for the three age ranges: 20–30, 30–45, and 45–60 yr.

### Displacement of the Thoracic Spine With Respect to the Sternum and Manubrium

Table 4 displays the results of measurements assessing the relationship between the thoracic spine and the sternum during breathing. As with the thoracic ROM, these measurements revealed statistically significant differences between inspiration and exhalation in all evaluated parameters.

**Table 4:** Mobility of the thoracic spine with respect to the sternum and manubrium.

	**Inspiration**		**Exhalation**		**Change**		**Wilcoxon signed-rank test**	
	**Mean ± SD**	**Range**	**Mean ± SD**	**Range**	**Mean ± SD**	**Range**	**Z**	***p***
Sternum T1–T2 angle (degrees)	42.9 ± 8.1	30–62	48.8 ± 8.4	35–69	5.8 ± 2.0	2–9	−5,429	0.000
Manubrium T1–T2 angle (degrees)	54.5 ± 7.9	40–67	58.9 ± 8.1	45–74	4.4 ± 2.1	2–8	−5,030	0.000
Distance LE sternum to T12 (mm)	181.4 ± 38.5	139.9–260.8	137.4 ± 38.5	81.2–223.5	43.8 ± 10.7	23–69	−5,373	0.000
LE sternum to T12 horizontal angle (degrees)	38.5 ± 7.5	26–55	25.3 ± 6.6	14–36	13.3 ± 5.4	0–25	−4,985	0.000
Distance UE manubrium to T1 (mm)	126.5 ± 15.5	99.5–158.5	121.2 ± 15.9	93.6–151.1	5.1 ±1.7	2–8	−5,112	0.000
UE manubrium T1 angle (degrees)	22.9 ± 6.4	11–37	41.4 ± 8.3	24–57	18.6 ± 6.4	4–32	−5,514	0.000
Distance T12 horizontal LE sternum	242.6 ± 25.5	196–284.6	196.1 ± 31.0	132.4–271.9	46.0 ± 16.8	18–80	−5,025	0.000

*LE, lower edge; UE, Upper edge*.

The angle formed by the body of the sternum and the anterior line of the T1 and T2 vertebral bodies increased during forced exhalation by 13.7% from the angle during inspiration. The distance from the inferior–posterior edge of the sternum to the T12 centroid decreased during forced exhalation by an average of 43.8 ± 10.7 mm (24%). The angle between the horizontal plane and the line joining the inferior–posterior edge of the sternum and the T12 centroid decreased significantly during exhalation by an average of 13.3° ± 5.4° (34.5%). The distance from the upper anterior edge of the sternum to the T1 centroid decreased during exhalation by a mean of only 5.1 ± 1.7 mm (4%), showing again that this upper aspect of the thorax is quite rigid.

During forced exhalation, there was an inferior displacement of the upper aspect of the thorax, as the angle of the line from the upper anterior edge of the sternum to the T1 centroid increased by an average of 18.6° ± 6.4° (80.8%) in relation to the horizontal plane. This descent of the thorax during exhalation was also shown by a decrease of 18.9% in the distance between the T12 centroid and the horizontal line starting at the front–superior edge of the sternum (Table 4).

## Discussion

The respiratory dynamics of the thoracic spine during forced ventilation were analyzed in this study. The results showed quantitatively that the kyphosis of the thoracic spine decreases during forced inspiration but increases during forced exhalation. However, an important finding of this study was that the three analyzed sectors of the thoracic spine showed a non-uniform mechanical behavior during breathing. The upper T1–T7 thoracic segment was more rigid than the T7–T10 segment. This different biomechanical behavior with increased mobility of the T7–T10 could be the underlying cause of the cranial displacement of the apex during forced exhalation.

The non-uniform sagittal ROM of the thoracic spine during deep breathing could be related to the different types of anatomic rib connections in the thoracic cage. At T1–T7 (the true ribs), the attachment of the ribs to the sternum and spine could greatly limit the mobility of the thoracic spine. A little mobility is expected, however, as the rib attaches to the cartilage before it attaches to the sternum. Ribs joined to T8–T10 (the false ribs) have longer costal cartilage, which attaches to other cartilage, resulting in more mobility in this area of the rib cage. At T11–T12 (floating ribs), the ribs have no anterior attachment, therefore, the greatest mobility of the thoracic spine can be expected in this area.

To date, some few studies have examined the impact of the rib cage on thoracic spine motion and stiffness (10–13). Using cadaveric models devoid of dynamic and stabilizing abdominal and paraspinal muscles, it has been found that the rib cage increases stiffness and decreases range of motion of the whole thoracic spine. Applying 400 N follower loads under ± 5 Nm dynamic moments to cadaveric specimens (13), the flexion/extension range of motion of the entire thoracic spine (T1–T12) was almost similar that found in the current *in-vivo* study. However, the ROM of the upper, middle, and lower segments of the thoracic spine found in specimens was different than in the current study on humans. One reason is that cadaveric specimens do not take into consideration the attachments of the upper thoracic spine to the cervical spine and scapulo-humeral joints. Similarly, at the lower thoracic spine, cadaveric specimens are usually devoid of the powerful abdominal stabilizing muscles, ileo-psoas and posterior paraspinal muscles. Although, models based on cadaveric specimens have tried to mimic the movements of the thoracic spine *in vivo*, all of them have still limitations the most important the arbitrary division of the thoracic spine in sectors that do not consider the anatomic characteristics of the thoracic spine (three types of rib attachments).

To our knowledge, there is only one study reporting normal functional ROM of the thoracic spine in alive subjects (5). A short ROM from T2–T3 to T6–T7 was found, very similar to our results. However, from T8–T9 to lower levels the ROM increased until a maximum of 4.2° at T12–L1. The discrepancy between these findings and the results of our study may be explained by the different position of the subject and the mechanical conditions applied. In our study, volunteers were standing position and only a maximal inspiration and exhalation under gravity was required for analysis of the thoracic ROM. In the previous study, participants were in supine position, and extension and flexion were forced by inserting triangular pillows below the back. The subjects were not affected by gravity, by stability of muscles, and for obese individuals, flexion and extension were more difficult. In addition, most of the participants had been diagnosed with cervical or lumbar spinal diseases although the thoracic spine was disease-free. Therefore, our results are not fully comparable and represent the ROM of the thoracic spine during breathing with more reliability.

Notably, this work demonstrates that the most mobile thoracic segment during forced deep breathing is T7–T10, the false rib region. The movement of this short segment provides enough expansion for respiratory function in cases demanding high ventilation rates. The T7–T10 inspiratory lordosis facilitates the ascension and expansion of the thoracic cage when deep breathing is required. The increase in lordosis could also facilitate the function of the intercostal muscles and diaphragm. Concerning the ROM of the discs corresponding to the T7–T10 segment, our findings are in accordance with those reported by White and Panjabi (14) estimated a representative ROM of 6 degrees for T7–T8 through T9–T10. Our measurements in the T7–T10 are similar (12 degrees). These authors also suggests that respiration utilizes most of the available ROM in this region.

The rather short ROM of the T10–T12 vertebral segment, which is almost fixed during peak respiratory movements, was unexpected, considering that this segment represents the area of greatest mobility in the thoracic spine. This segment is located in the transition zone between the rigid thoracic and mobile lumbar spine, which supports high mechanical stress. This vertebral segment is not kyphotic or lordotic on the sagittal plane.

Displacement of the thoracic spine in relation to the sternum was also evaluated in this study. The results again demonstrated the rigidity of structures located at the upper level of the respiratory bellows: the sternum, the two first ribs, and discs T1–T2. The distance from the T1 vertebra to the sternum changed minimally during maximum inspiration and exhalation, while the distance from the T12 vertebra to the sternum changed markedly.

Together, these data provide novel implications for the role of the thoracic spine in respiratory mechanics. To date, the literature has attributed only a passive role to the thoracic spine in respiratory function. The respiratory motor is mainly believed to be the diaphragm, the intercostals and other accessory muscles (2, 15). These primary respiratory muscles have never included the thoracic spine erector muscles. However, breathing dynamics is highly dependent on the movements of the thoracic spine in extension that is attributable to the contraction of the paraspinal extensor muscles. These muscles have a definite relevant role in respiratory function particularly when maximal breathing is required.

The thoracic spine erector muscles are responsible for the lordotic movements of the T7–T10 segment, which triggers chest elevation and increases the respiratory exchange surface on all planes. Thus, the inspiratory lordotic and the expiratory kyphotic effect occurs in the most elastic segment of the thoracic spine at the base of the thorax, where the range of motion of the lever arm in relation to the sternum is greater, which maximizes the effects of spinal lordosis and kyphosis on the breathing response.

The thoracic spine not only has the ability to maintain chest height but also seems to be the primary respiratory longitudinal engine for forced inspiration. In addition, the thoracic spine preserves respiratory collapse during exhalation to the flexion limit and enables lung expansion during forced inspiration. The thoracic spine not only behaves in these situations as a facilitator of costal and diaphragmatic breathing but also directly increases the respiratory exchange surface during forced inspiration. The inspiratory spinal lordosis observed in our study enables chest expansion, horizontalization of the arch ribs, and expansion of the more elastic base of the thoracic cage. All of these mechanisms subsequently increase the respiratory exchange surface. The ascent of the upper rigid segment (the sternum, the first ribs, and the T1–T2 vertebral segment) induced by the inspiratory lordotic attitude of the thoracic spine also facilitates the expansion of the base of the chest by posteriorly displacing the T10–T12 segment. Furthermore, rib inspiratory expansion is predisposed to a more effective expiratory intercostal contraction, hence, decreasing the exchange surface at that time (11, 16).

For all of these functions, the spine also requires strong stability, ensured by the isometric coactivation of the paraspinal muscles, including the erector spinae (17, 18). At the same time, these muscles all contract rhythmically to actively assist ventilation during forced breathing (19). The close relationship between ventilation and spine erector muscle activation has been demonstrated previously by a number of studies; however, its function has been limited only to stabilizing the spine and not contributing to active inspiration (20–22).

The engine system that represents the mid-lower thoracic spine (T7–T10) is certainly the mechanism implied in forced respiratory function, beyond tidal ventilation. In addition, the lordotic displacement of the thoracic spine facilitates the respiratory function of the diaphragm, relieving abdominal pressure and expanding the thoracic basement to facilitate muscle contractility.

Considering that situations of high respiratory demand are frequent during normal active life, and although the respiratory functional reserve must be very large, vertebral movement can be triggered by submaximal efforts, which are very common during everyday activities. Apparently, the thoracic spine is needed for maximum respiratory requirements but is not triggered by tidal ventilation in healthy subjects. In conditions of respiratory restriction, the thoracic spine mechanism could also be solicited at rest. The limited flexion-extension range of motion of the T7–T10 segment that is presumed to occur in spinal deformities, such as thoracic idiopathic scoliosis frequently showing the apex located within this segment, could be an additional factor implicated in the restricted respiratory function of these patients. Additionally, the abolition of T7–T10 motion in the sagittal plane conditioned by the current techniques for surgical management of the spinal deformities requiring segmental fusion could have severe implications on the respiratory function of such patients.

The present study might be influenced by certain methodological limitations. First, the forced respiratory maneuvers were voluntary and followed detailed instructions to the participating individuals. However, the maximal strength of the paraspinal muscles during inspiration was never controlled. Standardization of maximal exhalation was also challenging, and both extension and flexion of the thoracic spine were especially difficult in the most obese individuals. Another limitation is that the findings may not generalize to older adults or, obviously, to those with spine conditions. In addition, pulmonary functional tests were not performed to the participants. Retrospectively, a correlation of thoracic ROM during breathing with baseline ventilation data would be worthy. Considering these limitations, we observed the same pattern of thoracic and sterno-thoracic displacement in all individuals independently of gender and age. In addition, this is the first study to address the ROM of the thoracic spine during respiratory mechanisms.

In conclusion, this study provides new insights into the dynamic behavior of the thoracic spine during respiratory function. Until now, the thoracic spine has been considered rather stable due to its restriction by the rib cage. However, the results of this study demonstrate that the thoracic spine plays a primary role during respiratory function, being the longitudinal driving force in the chest in situations requiring maximal breathing. The key issue is that maximal inspiration is highly dependent on the angular movement of the T7–T10 segment, corresponding to the floating ribs. The sternum, the first shorter ribs, and the first intervertebral discs are less elastic and can be considered passive elements. These findings are important when considering the clinical implications that thoracic fusion might have for patients with spinal deformities requiring surgery.

## Data Availability Statement

The original contributions presented in the study are included in the article/supplementary files, further inquiries can be directed to the corresponding author/s.

## Ethics Statement

The studies involving human participants were reviewed and approved by the Institutional Review Board of the Hospital Ramón y Cajal (Madrid, Spain) with protocol number: V1-08/05/2016. The patients/participants provided their written informed consent to participate in this study.

## Author Contributions

All authors listed have made a substantial, direct and intellectual contribution to the work, and approved it for publication.

## Conflict of Interest

The authors declare that the research was conducted in the absence of any commercial or financial relationships that could be construed as a potential conflict of interest.

## Publisher's Note

All claims expressed in this article are solely those of the authors and do not necessarily represent those of their affiliated organizations, or those of the publisher, the editors and the reviewers. Any product that may be evaluated in this article, or claim that may be made by its manufacturer, is not guaranteed or endorsed by the publisher.
